# Kinetics of Physiological and Behavioural Responses in Endotoxemic Pigs with or without Dexamethasone Treatment

**DOI:** 10.3390/ijms20061393

**Published:** 2019-03-20

**Authors:** Zhiwei Li, Ellen Kanitz, Margret Tuchscherer, Armin Tuchscherer, Cornelia C. Metges, Nares Trakooljul, Klaus Wimmers, Eduard Murani

**Affiliations:** 1 Institute of Genome Biology, Leibniz Institute for Farm Animal Biology (FBN), Wilhelm-Stahl-Allee 2, 18196 Dummerstorf, Germany; li.zhiwei@fbn-dummerstorf.de (Z.L.); trakooljul@fbn-dummerstorf.de (N.T.); wimmers@fbn-dummerstorf.de (K.W.); 2 Institute of Behavioural Physiology, Leibniz Institute for Farm Animal Biology (FBN), Wilhelm-Stahl-Allee 2, 18196 Dummerstorf, Germany; ellen.kanitz@fbn-dummerstorf.de (E.K.); mtuchsch@fbn-dummerstorf.de (M.T.); 3 Institute of Genetics and Biometry, Leibniz Institute for Farm Animal Biology (FBN), Wilhelm-Stahl-Allee 2, 18196 Dummerstorf, Germany; atuchsch@fbn-dummerstorf.de; 4 Institute of Nutritional Physiology “Oskar Kellner”, Leibniz Institute for Farm Animal Biology (FBN), Wilhelm-Stahl-Allee 2, 18196 Dummerstorf, Germany; metges@fbn-dummerstorf.de

**Keywords:** dexamethasone, lipopolysaccharide, pig, endotoxemia, physiological responses, sickness behaviour

## Abstract

Although dexamethasone (DEX) is a widely used immunoregulatory agent, knowledge about its pharmacological properties in farm animals, especially pigs, is insufficient. Previous studies suggest that compared to other species, pigs are less sensitive to the immunosuppression conferred by DEX and more sensitive to the threat of bacterial endotoxins. However, there is a paucity of studies examining DEX immunomodulation in endotoxemia in this species. In this study, a porcine endotoxemia model was established by lipopolysaccharide (LPS) and the effect of DEX-pretreatment on the magnitude and kinetics of neuroendocrine, metabolic, hematologic, inflammatory, and behavioural responses were examined. DEX decreased cortisol, adrenocorticotropic hormone (ACTH), red blood cell, hemoglobin, hematocrit, and lymphocyte whereas glucose concentration was increased under both normal and endotoxemic conditions. By contrast, DEX decreased triglyceride, lactate, and IL-6 concentrations and increased platelet count only under an endotoxemic condition. DEX also reduced the frequency of sickness behaviour following LPS challenge. PCA showed that glucose and triglyceride metabolism together with red blood cell count mainly contributed to the separation of clusters during DEX treatment. Our study demonstrates that DEX protects pigs from inflammation and morbidity in endotoxemia, in spite of their less sensitivity to DEX. Moreover, its considerable role in the regulation of the metabolic and hematologic responses in endotoxemic pigs is revealed for the first time.

## 1. Introduction

Glucocorticoids (GCs) are a class of steroid hormones produced and secreted by the adrenal cortex as the final output of the neuroendocrine hypothalamus-pituitary-adrenal (HPA) stress axis [1]. They orchestrate many physiological activities, like metabolism and immune response, to maintain physiological equilibrium. GCs repress the expression of numerous inflammatory genes, including those encoding pro-inflammatory cytokines (e.g., TNF-α, IFN-γ, IL-1, IL-6) via glucocorticoid receptor (GR) [2]. Pro-inflammatory cytokines released from activated monocytes and macrophages, e.g., in response to endotoxins, in turn strongly activate the HPA axis and ultimately stimulate the production of GCs to prevent overshooting of the inflammatory responses [3]. Owing to their potent anti-inflammatory action GCs became the most widely prescribed drugs in the world. A variety of compounds with GC activity has been developed and clinically used in humans in the therapy of various inflammatory and autoimmune diseases [1]. Likewise, GC-based drugs also play an irreplaceable role in veterinary medicine [4]. 

As one of the most potent GC drugs, dexamethasone (DEX) is extensively applied for the treatment of inflammatory and immunological dysfunctions [5,6]. In addition, considering the comparatively rapid clearance in pigs [7], DEX seems to be a promising agent to protect pigs from the threat of systemic inflammation induced by various infections and to improve growth performance under different stress conditions like weaning by modulating the immune system [8,9]. Although the pharmacological effects of DEX on inflammation were comprehensively studied in humans and model animals [2,10,11], related information in many important farm animals, especially pigs, is relatively insufficient [5]. Thus, GC therapy in most domestic animal species relies on clinical experience and knowledge from human medicine [4]. Compared to other mammalian species like cattle, pigs are relatively resistant to immunosuppression conferred by DEX manifested by less sensitivity and persistence of the response of antibody and immune cells to DEX in the latter [12,13]. This relative resistance results, at least partly, from faster DEX metabolism in pigs compared to dogs, cattle, horses and humans [7]. Hence, it is apparently inappropriate to use DEX in pigs based on the knowledge from other species including humans. In this context, the overall effect of DEX on general physiological indices includes not only immune/inflammatory, but also neuroendocrine, metabolic, and hematological variables that need comprehensive exploration to avoid unwanted side-effects.

Besides less DEX sensitivity pigs are more vulnerable to various inflammatory stimuli including bacterial endotoxins (e.g., lipopolysaccharide, LPS) [14]. Although LPS endotoxemia is the commonly used immune challenge in pigs [15], little is known about the anti-inflammatory effects of GCs in this context [5]. Given that pigs exhibit less sensitivity to DEX and conversely relatively greater sensitivity to endotoxins, it is imperative to reevaluate the specific effects of DEX on the endotoxin-induced inflammatory response in pigs.

As a synthetic hydrocortisone analogue, physiological action of DEX is tightly associated with feedback regulation of the hypothalamic-pituitary-adrenal gland (HPA) axis [1], which could be influenced by many factors like (epi)genetic make-up including genotype of GR and health condition, resulting in a large individual variation in therapeutic effects conferred by GCs [16,17,18]. DEX is widely used to examine individual differences in HPA feedback regulation under non-inflammatory condition (DEX suppression test) [16], but under inflammatory condition this test was so far limited to model animals [19]. However, HPA axis regulation in response to immune challenges is an important facet of stress resilience; a concept attracting increasing attention for its clear health benefits [20].

Therefore, the aim of the present study is to contribute to the knowledge base for therapeutic as well as the research application of DEX in pigs by exploring DEX action in a porcine endotoxemia model established by LPS. The effects of DEX under basal condition and on LPS-induced inflammatory responses were examined by multiple biomarkers. During this process, sickness behaviour and kinetics of neuroendocrine, hematological, metabolic, and inflammatory responses were also monitored.

## 2. Results

**Figure 1:**
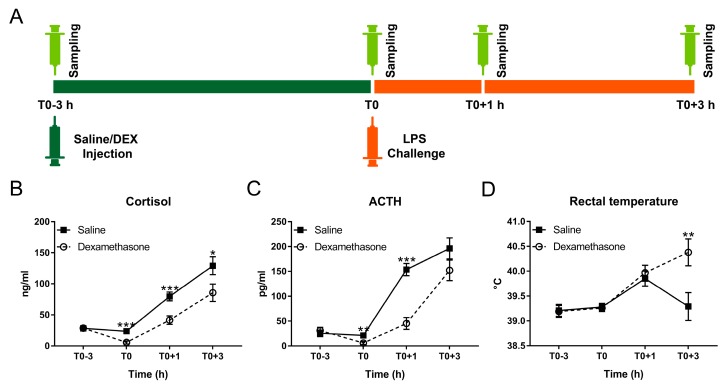
Effect of dexamethasone (DEX) on febrile and neuroendocrine responses following lipopolysaccharide (LPS) challenge. (**A**) Schematic diagram of experimental design; (**B**) cortisol; (**C**) adrenocorticotropic hormone (ACTH); and (**D**) rectal temperature. Data are presented as least-squares means ± SE. * *p* < 0.05, ** *p* < 0.01, *** *p* < 0.001.

To comprehensively explore the effect of DEX on LPS-induced physiological and behavioural responses, pigs pretreated with DEX or saline were challenged with a dose of LPS that has been previously described [15] to induce systemic inflammation (Figure 1A). Results of statistical analysis are summarized in Table 1 and Table S1 for behavioural and physiological responses, respectively, and described in more detail below.

### 2.1. DEX Diminished LPS-Induced Neuroendocrine Response

Feedback regulation of the HPA axis during acute challenges is an important factor determining the extent of the physiological disturbances and duration of recovery [21]. Thus, we measured DEX-mediated suppression of the HPA axis in both non-inflammatory and endotoxemic pigs.

At T0, DEX almost totally blocked the production of cortisol and adrenocorticotropic hormone (ACTH) under non-inflammatory condition (Figure 1B,C). By contrast, LPS triggered pronounced cortisol and ACTH responses with dissociated kinetics in all challenged pigs (Figure 1B,C; Table S1). During inflammation, cortisol and ACTH concentrations in the DEX group were less than that in the saline group but still showed a notable increase compared with basal concentrations (Figure 1B,C; Table S1).

LPS caused an obvious febrile response in all pigs at T0+1 h, which continuously became stronger in the DEX group until at least T0+3 h (Figure 1D; Table S1). Conversely, the rectal temperature of saline-pretreated pigs decreased from T0+1 to T0+3 h (Figure 1D; Table S1).

### 2.2. DEX Altered Glucose and Triglyceride Metabolism during Inflammation

Considering that the response of animals to infection is usually accompanied by anorexia and disturbance in metabolism, resulting in impeded growth and degraded product quality [22], we explored if DEX could improve metabolic health during the acute inflammatory response.

**Figure 2:**
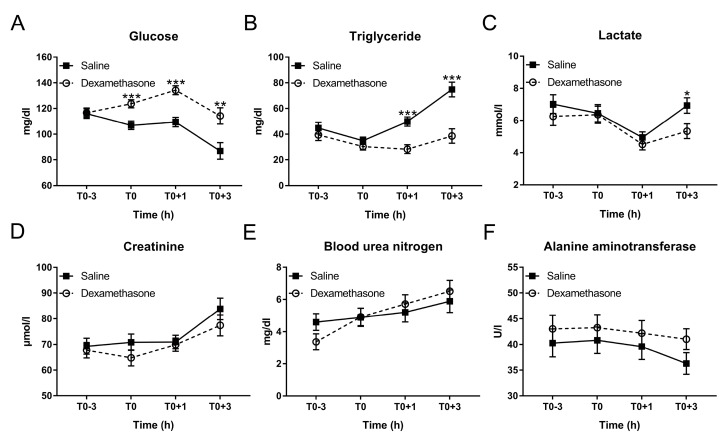
Effect of DEX on the kinetics of plasma biochemical parameters following LPS challenge. (**A**) Glucose; (**B**) triglyceride; (**C**) lactate; (**D**) creatinine; (**E**) blood urea nitrogen (BUN); and (**F**) alanine aminotransferase (ALT). Data are presented as least-squares means ± SE. * *p* < 0.05, ** *p* < 0.01, *** *p* < 0.001.

As expected, at T0, DEX elevated glucose concentration, but had no effect on triglycerides under non-inflammatory condition (Figure 2A,B). After LPS challenge, saline-pretreated pigs exhibited a significant decrease in glucose concentration from T0+1 to T0+3 h and increase in triglyceride from T0 to T0+3 h, which were prevented by the pretreatment of DEX (Figure 2A,B; Table S1).

Lactate concentration dropped significantly in both groups at T0+1h and raised again at T0+3h, reaching significantly greater concentrations in the saline-pretreated group (Figure 2C; Table S1).

In addition, LPS elevated the concentration of creatinine and blood urea nitrogen (BUN), which were not reversed by DEX (Figure 2D,E; Table S1). Both LPS and DEX showed no obvious effect on the concentration of alanine aminotransferase (ALT) (Figure 2F; Table S1).

### 2.3. DEX Blocked LPS-Induced Hematological Response in Varying Degrees

**Figure 3:**
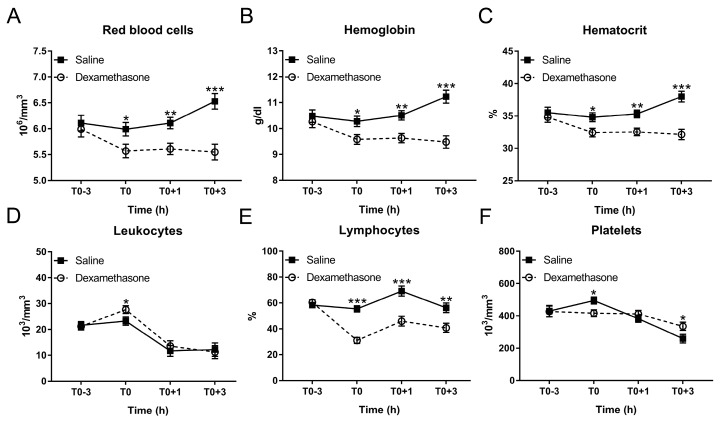
Effect of DEX on the kinetics of hematological parameters following LPS challenge. (**A**) Red blood cell count; (**B**) hemoglobin concentration; (**C**) hematocrit; (**D**) leukocyte count; and (**E**) lymphocyte proportion; (F) platelet count. Data are presented as least-squares means ± SE. * *p* < 0.05, ** *p* < 0.01, *** *p* < 0.001.

Hematological disturbances are closely associated with the clinical situation and are also useful indicators for the poor growth performance in pigs [23]. Hence, we determined if DEX contributes to hematological integrity in endotoxemic pigs. At T0, DEX reduced red blood cells, hemoglobin and hematocrit under non-inflammatory condition (Figure 3A–C). After LPS challenge, saline-pretreated pigs exhibited a significant increase in these variables at T0+3 h, which could be completely blocked by DEX-pretreatment (Figure 3A–C; Table S1).

In contrast, although DEX increased leukocytes at T0, it was unable to reverse LPS-induced leukocyte decrease (Figure 3D; Table S1). After LPS challenge, lymphocyte proportion in the saline group was increased at T0+1 h and returned to the baseline at T0+3 h (Figure 3E; Table S1). DEX reduced lymphocyte proportion under non-inflammatory condition; and this remained significantly less until T0+3 h (Figure 3E; Table S1).

Endotoxemia is frequently accompanied by severe thrombocytopenia [24]. We found that LPS decreased platelet number in a time-dependent manner, which could be partially reversed by DEX (Figure 3F; Table S1).

### 2.4. DEX Relieved LPS-Induced Systemic Inflammation

**Figure 4:**
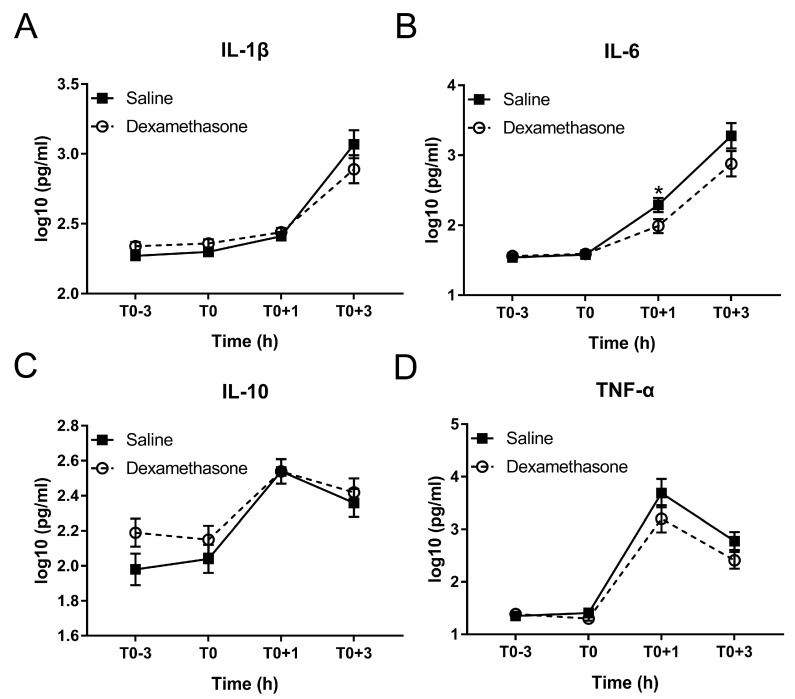
Effect of DEX on the inflammatory response following LPS challenge. (**A**) IL-1β; (**B**) IL-6; (**C**) IL-10; and (**D**) TNF-α. Data are presented as least-squares means ± SE. * *p* < 0.05.

Excessive production of inflammation-associated cytokines is a hallmark of endotoxemia [25]. Therefore, we measured four cytokines including pro-inflammatory IL-1β, IL-6, TNF-α, and their counterplayer IL-10 during the whole experiment. DEX showed no obvious effect on the production of these four cytokines under non-inflammatory condition (Figure 4A–D).

After LPS challenge, IL-1β and IL-6 were increased in a time-dependent manner in both groups until at least T0+3 h (Figure 4A,B; Table S1). Pretreatment by DEX did not alter the kinetics but reduced the concentration of IL-1β and IL-6 during inflammation: IL-6 concentration in the DEX group was significantly less than that in the saline group at T0+1 h; although the difference did not reach statistical significance, both IL-1β and IL-6 concentrations in DEX group were numerically less than that in saline group at T0+3 h (Figure 4A,B).

The kinetics of IL-10 and TNF-α was different from that of IL-1β and IL-6. Both peaked at T0+1h and declined from T0+1 to T0+3 h (Figure 4C,D; Table S1). Although showing no effect on the LPS-induced kinetics of TNF-α, DEX numerically reduced its concentration at both T0+1 h and T0+3 h (Figure 4D). Conversely, DEX showed no notable effect on IL-10 (Figure 4C).

### 2.5. DEX Alleviated LPS-Induced Sickness Symptoms

Elevated pro-inflammatory cytokines are associated with clinical symptoms and distinct behavioural changes, including lethargy and social withdrawal [26], summarily designated as sickness behaviour. To evaluate the effect of DEX on LPS-induced behavioural responses, the frequency of five sickness symptoms over a period of 4 h post LPS challenge was recorded and summarized in Table 1.

The most remarkable effect of DEX was pronounced reduction of panting. While in the saline group the frequency of panting peaked during the 2nd h, in the DEX group it was steadily decreasing and overall significantly less, particularly during 2nd, 3rd, 4th h post challenge (*p* < 0.001, < 0.001, = 0.030, and = 0.045, respectively). In addition, in the DEX group also the overall occurrence of cyanosis was reduced. The frequency of cyanosis in both groups peaked during the 4th h post LPS challenge with a tendency for a reduced peak value in the DEX group (3.11 ± 0.64 vs. 0.38 ± 0.65). Within the total time of observation, the average frequency of cyanosis in the DEX group was significantly less than that in the saline group (*p* = 0.024).

No statistically significant difference was observed in vomiting and inactivity between the two groups. DEX group showed a tendency for reduced vomiting and inactivity as manifested by the delayed arrival of peak and numerically decreased peak value. In saline and DEX groups, frequency of vomiting peaked during the 1st h and 2nd h post LPS challenge respectively with a numerically less peak value in the latter (0.78 ± 0.14 vs. 0.43 ± 0.15); similarly, the frequency of inactivity peaked during the 2nd h and 3rd h post LPS challenge respectively with a numerically less peak value in the latter (11.72 ± 0.29 vs. 11.39 ± 0.30).

Different from other symptoms, DEX group showed a tendency for greater shivering as manifested by the greater peak frequency and the average frequency compared to that in the saline group, but the difference did not reach statistical significance.

### 2.6. The Overall Effect of DEX on Physiological Responses in Normal and Endotoxemic Pigs

Finally, we performed principal component analysis (PCA) to determine the contribution of physiological variables to the effect of DEX under both normal and endotoxemic condition. LPS shifted all challenged pigs regardless of pretreatment in the same direction featured by the increase in cortisol, ACTH, and IL-6, and the decrease in leukocytes and platelets, which exhibited a growing variation among individuals as indicated by the continuously decentralized symbols (Figure S1).

**Figure 5:**
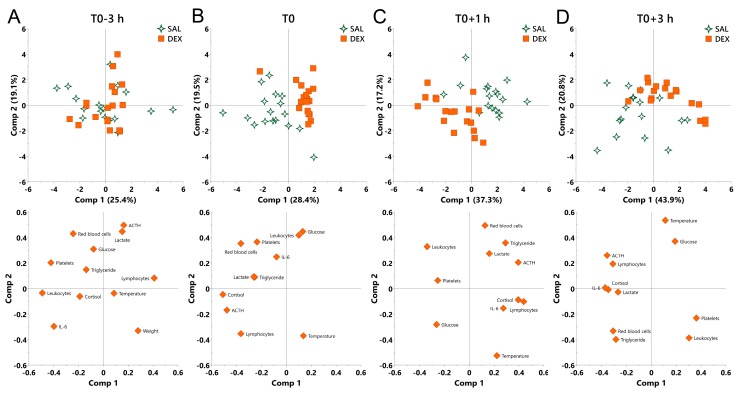
Principal component analysis (PCA) for the overall effect of DEX on pigs under normal and endotoxemic conditions. (**A**) T0-3 h, baseline; (**B**) T0, the effect of DEX under normal condition; (**C**) T0+1 h and (**D**) T0+3 h, the effect of DEX under endotoxemic condition. Loading of the parameters on component 1 and 2 are visualized below the PCA score plot to show the contribution of parameters to the group separation. SAL, saline; DEX, dexamethasone; Comp, component.

At the beginning of the experiment (T0-3 h), no discrimination was observed between saline and DEX groups (Figure 5A). At T0, DEX shifted pigs to the direction along the decrease in cortisol and ACTH and overall compressed the individual variation as reflected by the centralized symbols (Figure 5B).

During inflammation, a notable discrimination was observed between saline and DEX groups (Figure 5C,D). At T0+1 h, PC1 was mainly driven by an opposition between cortisol, ACTH, and lymphocytes (negatively correlated with DEX-induced shift), and leukocytes (positively correlated with DEX-induced shift), whereas PC2 was mainly driven by an opposition between red blood cells and triglycerides (negatively correlated with DEX-induced shift), and rectal temperature (positively correlated with DEX-induced shift) (Figure 5C). At T0+3 h, PC1 was mainly driven by an opposition between cortisol, ACTH, and IL-6 (negatively correlated with DEX-induced shift), and platelets (positively correlated with DEX-induced shift), whereas PC2 was mainly driven by an opposition between red blood cells, triglyceride and leukocytes (negatively correlated with DEX-induced shift), and rectal temperature and glucose (positively correlated with DEX-induced shift) (Figure 5D). During the whole inflammatory process, DEX consistently shifted pigs to the direction along the increase in glucose and the decrease in red blood cells and triglycerides (Figure 5C,D).

## 3. Discussion

To obtain a comprehensive overview of DEX effects in pigs we analyzed diverse biological responses in resting and LPS-challenged animals.

Compared to poultry and rodents, pigs are relatively sensitive to LPS so that doses in excess of 25 μg/kg BW are considered as high [14]. This may explain a decrease in rectal temperature from T0+1 to T0+3 h in the saline group since sometimes decrease in temperature could be observed in animals mounting strong inflammatory response [27].

Although DEX almost totally blocked the release of cortisol and ACTH at T0 due to the negative feedback of glucocorticoids on their own secretion [28], it only partially reduced LPS-triggered neuroendocrine responses. In fact, pro-inflammatory cytokines induce tissue-specific glucocorticoid insensitivity by interfering with glucocorticoid receptor signaling [3]. While reduced glucocorticoid sensitivity in the HPA axis may serve as an adaptive mechanism facilitating stronger or prolonged glucocorticoid release, in peripheral tissues it has adverse effects and may lead to sepsis [1,3]. We found that the kinetics of the cortisol response was dissociated from that of ACTH during the experiment. LPS-induced cytokines like IL-1β, IL-6, and TNF-α can either directly promote the release of cortisol by interacting with the adrenal gland, or function indirectly by interacting with hypothalamus and pituitary, resulting in the release of corticotropin-releasing hormone (CRH) and ACTH and thereby increase cortisol via activation of the HPA axis [29]. It appears that sensitivity to both, LPS and DEX, differs between the central branch of the HPA axis and the adrenal gland, thus pointing to different regulation of their responses.

Glucose was sharply reduced from T0+1 to T0+3 h accompanied by an increase in lactate, which may be explained by the anaerobic glycolysis during endotoxemia. It has been described that LPS promotes glycolysis via enhancing the expression and the activity of glycolysis-associated enzymes such as hexokinase, pyruvate kinase, and lactic dehydrogenase [30]. Then the product of glycolysis, pyruvate, could be converted to lactate due to the hypoxia which normally occurs in LPS-induced endotoxemia [31,32]. Notably, a decrease in lactate was observed in both groups at T0+1 h. This may be associated with the removal of feed/anorexia during the experiment since a recent study demonstrated that circulating lactate could also be a primary source of carbon for the tricarboxylic acid cycle under fasting condition for hepatic gluconeogenesis [33]. Importantly, the subsequent rise in lactate at T0+3 h was blunted by DEX. This is a significant beneficial effect of DEX in endotoxemia, since lactate accumulation may lead to lactic acidosis, which is a serious complication in sepsis [1].

An obvious distinction in lipid metabolism was observed between saline and DEX groups. Whereas LPS has been shown to rapidly induce changes in hepatic lipid metabolism leading to hypertriglyceridemia [34], glucocorticoids including DEX induce lipolysis in adipose tissue [35], particularly during fasting [36].

An increase in creatinine and BUN was observed after LPS challenge, which was supported by similar studies using mouse or pig model [37,38]. In the study using C57BL/6 mice, the increase in creatinine and BUN was partially inhibited by pretreatment of DEX 1 h before LPS injection [37]. However, in our experiment DEX showed no effect on LPS-induced increase in creatinine and BUN, which may be associated with experimental factors and/or species differences.

We found ALT, the specific marker for hepatic injury, was slightly decreased at T0+3 h. This result is consistent with a previous observation that in pigs LPS increased the nonspecific hepatic injury marker aspartate aminotransferase (AST) and the ratio of AST/ALT at 3 h post challenge but showed no obvious effect on ALT concentration [39].

DEX decreased red blood cell related properties (number, blood hemoglobin content, and hematocrit) irrespective of the LPS treatment. Conversely, LPS increased these properties at T0+3 in saline-treated animals. This effect of DEX on red blood cells has not been characterized in pigs so far. Nevertheless, it is supported by the study of Sautron et al. [40], who observed a similar decrease in red blood cells following ACTH application, stimulating cortisol production. The mechanism behind this effect in pigs warrants further investigation. This could be related to the effect of glucocorticoids on fluid homeostasis or haemodynamics [4]. The decrease in the total number of leukocytes following LPS challenge might be, at least partly, associated with adhesion and migration of neutrophils [41]. Neutrophils are one of the most abundant leukocytes, which account for more than 40% of total leukocytes [42]. LPS could enhance the expression of endothelial adhesion molecules and thus promote the adhesion of neutrophils to endothelial cells and the transendothelial neutrophil migration, leading to the reduction of neutrophils in peripheral blood [41]. Although DEX could inhibit the migration of neutrophils by up-regulating annexin A1 via glucocorticoid-induced leucine zipper [43], it failed to reverse LPS-induced reduction in total circulating leukocytes. In contrast, DEX increased circulating leukocytes under the non-inflammatory condition, which may be explained by the increase in neutrophils, since the administration of glucocorticoids could increase neutrophils via enhancing neutrophil demargination from the endothelial layer and neutrophil release from the bone marrow [44].

We found that DEX blunted LPS-induced thrombocytopenia, a hallmark of sepsis and endotoxemia [45]. Platelet activation and local coagulation is a protective mechanism against endothelial dysfunction [46], and against pathogens serving their trapping and elimination [47]. However, exaggerated platelet activation is a major contributor to thrombocytopenia and may lead to disseminated intravascular coagulation, and ultimately to multiple organ failure [47]. Thus, prevention of thrombocytopenia is another important aspect contributing to the protective effect of DEX in endotoxemia, likely by inhibiting platelet aggregation [48].

So far, only a single study reported on DEX effects on inflammatory responses, including cytokine production, to LPS application in pigs [5]. However, the route of application (intravenous), dose of both LPS and DEX, application scheme of DEX, and LPS serotype (O55:B5) were different from the present study. Nevertheless, the results for IL-6, and IL-10 were similar. More specifically, whereas IL-6 was most potently reduced by DEX, while IL-10 activation was not influenced by DEX [5]. Similar to the study of Myers et al. [5], in our study TNF-α was also increased at 1 h post LPS challenge. However, the statistically significant effect of DEX on this increase was not observed in current study. A notable difference observed here is activation of IL-1β by LPS, which in the study of Myers et al. [5] was constitutively present and unaffected by both LPS and DEX. This dichotomy is likely explained by differences in LPS serotype and/or application.

Using PCA we found that LPS significantly shifted the clusters in a time-dependent manner regardless of the DEX treatment, which was mainly driven by the responses of neuroendocrine and immune systems, together with changes in platelet number. This observation is consistent with previous reports that LPS-induced endotoxemia is usually accompanied by systemic inflammation and thrombocytopenia [49,50]. In addition, we found that the LPS-induced excessive physiological responses amplified the intrinsic individual variation which was manifested by dispersal of the clusters in a time-dependent manner. This observation is supported by previous studies likewise reporting tremendous variation among individuals in physiological responses to LPS [5,51]. Similar to LPS, DEX also significantly shifted PCA clusters and this effect persisted both under inflammatory and non-inflammatory conditions. Notably, although LPS significantly affected triglyceride metabolisms, this was not the primary factor contributing to LPS-induced overall alteration. In contrast, the changes in glucose and triglycerides played an important role in DEX-induced overall shift during inflammation, implying that energy transformation may be an additional important factor for DEX-mediated protective effect in endotoxemic pigs [52].

## 4. Materials and Methods

### 4.1. Animals, Combined DEX/LPS Challenge, and Sampling

Seven-week-old (n = 36, 18 females and 18 males) purebred German Landrace pigs were used in this study. The experiment was performed in three replicates each consisting of 12 animals (6 males and 6 females). The pigs were born and reared at the experimental pig farm of the Leibniz Institute for Farm Animal Biology (FBN) (Dummerstorf, Germany) under standardized conditions. Before entering the experiment their health status was visually approved by trained personnel. The following criteria were used for the visual approval: general condition, lethargy, refusal to eat, consistency of faeces, skin discolouration, coughing, lameness, swelling on the body or joints and abnormal behaviour. Two days before the experiment, the pigs were transferred to single pens (1.90 × 1.10 m^2^) to facilitate observation of sickness behaviour during the challenge. Each pen was equipped with a feeder and a nipple waterer. One day prior to the experiment all pigs were weighed (mean ± SE = 13.6 ± 0.3 kg). The pigs were assigned into two experimental groups, each balanced for sex (3 males and 3 females in each group per replicate; in total 9 males and 9 females per group): 1. DEX which was given a bolus intramuscular injection of 60 μg/kg BW DEX sodium phosphate (Dexatat, aniMedica, Senden, Germany) at T0-3 h (three hours before the LPS challenge; ~8:00 a.m.). 2. Saline which was given a bolus intramuscular injection of the corresponding volume of sterile, endotoxin-free 0.9% saline at T0-3 h. At T0 (three hours post DEX/saline administration), all pigs were intraperitoneally injected with 100 μg/kg BW LPS (Escherichia coli O111: B4; Sigma-Aldrich, Taufkirchen, Germany) as previously described [53]. During the experiment (starting at T0-3 h) all animals were deprived of feed.

Blood samples were obtained via rapid (≤ 30s) anterior vena cava puncture at T0-3 h, T0, T0+1 h, and T0+3 h and collected into pre-chilled EDTA tubes. In addition, rectal temperature was measured at the same timepoints to monitor the febrile response.

Experimental animal use, care, handling and sample collection were performed under European Union and German legislation of animal protection. The experimental protocol and procedures were approved by the Animal Care Committee of the Leibniz Institute for Farm Animal Biology and the State Mecklenburg-Western Pomerania (Landesamt für Landwirtschaft, Lebensmittelsicherheit und Fischerei; LALLF M-V/TSD/7221.3-1-024/16; approval date: 26 May 2016).

### 4.2. Measurement of Neuroendocrine Parameters

Plasma concentration of ACTH and cortisol were measured in duplicate using commercially available ELISA kits according to the manufacturer’s recommendations (DRG Instruments GmbH, Marburg, Germany). The kits (EIA 3647 for ACTH and EIA 1887 for cortisol) used for the measurement of porcine plasma were validated in our previous study [18].

### 4.3. Measurement of Biochemical Parameters

Plasma concentration of BUN, triglyceride, and glucose were detected by a Fuji DriChem 4000i clinical chemistry analyzer (Scil, Viernheim, Germany). Plasma concentration of lactate, creatinine, and ALT were measured by an enzymatic-spectrophotometric assay using an ABX Pentra 400 instrument.

### 4.4. Measurement of Hematological Parameters

An aliquot of the blood samples was used to measure the hematological parameters including red blood cell count, hemoglobin, hematocrit, leukocyte count, lymphocyte proportion, and platelet count by the ABX Pentra 60 instrument (Axon Lab, Reichenbach/Stuttgart, Germany). Remaining blood samples were centrifuged at 4 °C and 2000× *g* for 20 min to obtain the plasma samples [18] and collected plasma samples were stored at –80 °C for further use.

### 4.5. Measurement of Inflammation-Associated Cytokines

Plasma concentration of inflammation-associated cytokines including IL-1β, IL-6, IL-10, and TNF-α were determined in duplicate using porcine cytokine magnetic bead panel kits according to the manufacturer’s recommendations (Merck, Darmstadt, Germany). The data was collected by the MAGPIX^®^ instrument (Merck).

### 4.6. Behavioural Observation

The behaviour of each animal was observed using scan sampling [54] every 5 min over a period of 4 h after LPS application. At the time of observation, following symptoms of sickness were assessed for each animal: (1) panting (respiratory difficulties) (2) shivering (pigs laid on the floor or stood and displayed rapid, synchronous muscle contractions, frequently accompanied by piloerection) (3) vomiting (4) cyanosis (peripheral). Furthermore, (5) animal activity was characterized as motor active (walking, employment with pen equipment) or inactive (lying, sitting, standing without movement). All observations were carried out by a trained person who was blinded to the treatment of the piglets.

### 4.7. Statistical Analysis

Prior to statistical analysis data distribution was tested to ensure approximate normal distribution. One animal (saline group) was excluded from the analysis due to uncertainty about the received treatment. Data on cytokine concentrations were log transformed to achieve approximate normality.

The effect of DEX on physiological responses to LPS represented by blood parameters was analysed using repeated measures ANOVA implemented in the mixed procedure in SAS/STAT software (version 9.4, SAS Inc., Cary, NC, USA). The main effects included in the model were treatment (DEX or saline), timepoint (T0-3 h, T0, T0+1 h, and T0+3 h, respectively), and their interaction, with the pig as experimental unit. The unstructured block diagonal covariance structure was used for the repeated measurement analysis on the same pig. In addition, the data were adjusted for the effects of glucocorticoid receptor genotype (GR_Ala610Val_ [18], all three allele combinations), sex (male and female), and replicate (1-3) by fitting these as fixed effects in the model. Least-squares means (LS-means) and their standard errors (SE) were computed for the timepoint × treatment interaction and compared within timepoint and treatment, respectively, using the slice option. Tukey-Kramer adjustment was applied within slices.

The effect of DEX on LPS-induced sickness behaviour represented by frequencies of symptom occurrence was analysed by fitting a Poisson model using the GLIMMIX procedure in SAS/STAT software. The model included the fixed effects treatment, time (1st h, 2nd h, 3rd h, and 4th h after LPS application, respectively), genotype, sex, replicate (1–3) and their interactions. Additionally, LS-means and their standard errors were computed for each fixed effect in the models, and all pairwise differences between LS-means were tested using the Tukey–Kramer procedure.

The contribution of physiological parameters to the overall effect of DEX and LPS was determined by PCA using SIMCA 14.1 (Umetrics, Umea, Sweden). Parameters showing a significant difference between DEX and saline groups were included in the PCA analysis with the exception of hemoglobin and hematocrit, which overlapped with red blood cell count [40]. Body weight was included in the PCA analysis at T0-3 h to visualize the contribution of this intrinsic factor to the initial distribution of pigs.

## 5. Conclusions

Despite the less sensitivity of pigs to DEX the latter improves physiological and behavioural integrity in endotoxemic pigs. Our results suggest that glucose, lactate and triglyceride metabolism as well as red cell and platelet count play a notable role in the beneficial effect of DEX in this context. This study closes the knowledge gap in the application of DEX in pigs and presents the feasibility of glucocorticoids for the improvement of animal robustness in the face of immune stress.

## Figures and Tables

**Table 1:** The frequency of sickness symptoms in pigs following LPS challenge.

Symptoms	Hour after LPS	Saline ^1^	Dexamethasone ^1^	*p*-Value
Panting	1st hour	2.67 ± 0.79	1.08 ± 0.80	0.849
	2nd hour	6.29 ± 0.79	0.99 ± 0.80	< 0.001
	3rd hour	4.67 ± 0.79	0.96 ± 0.80	0.030
	4th hour	4.22 ± 0.79	0.68 ± 0.80	0.045
	Overall	4.46 ± 0.54	0.93 ± 0.61	< 0.001
Shivering	1st hour	2.22 ± 0.92	2.14 ± 0.94	1.000
	2nd hour	3.44 ± 0.92	6.47 ± 0.94	0.307
	3rd hour	4.33 ± 0.92	6.04 ± 0.94	0.896
	4th hour	3.44 ± 0.92	5.21 ± 0.94	0.879
	Overall	3.36 ± 0.63	4.97 ± 0.64	0.088
Vomiting	1st hour	0.78 ± 0.14	0.20 ± 0.15	0.099
	2nd hour	0.67 ± 0.14	0.43 ± 0.15	0.940
	3rd hour	0.11 ± 0.14	0.01 ± 0.15	0.990
	4th hour	0.01 ± 0.14	0.01 ± 0.15	1.000
	Overall	0.39 ± 0.08	0.16 ± 0.09	0.062
Cyanosis	1st hour	0.01 ± 0.64	0.01 ± 0.65	1.000
	2nd hour	1.39 ± 0.64	0.01 ± 0.65	0.789
	3rd hour	2.11 ± 0.64	0.01 ± 0.65	0.298
	4th hour	3.11 ± 0.64	0.38 ± 0.65	0.071
	Overall	1.65 ± 0.45	0.08 ± 0.46	0.024
Inactivity	1st hour	11.11 ± 0.29	10.42 ± 0.30	0.709
	2nd hour	11.72 ± 0.29	11.35 ± 0.30	0.985
	3rd hour	11.11 ± 0.29	11.39 ± 0.30	0.990
	4th hour	11.22 ± 0.29	11.29 ± 0.30	1.000
	Overall	11.29 ± 0.21	11.11 ± 0.21	0.548

^1^ Least-squares means ± SE.

## Supplementary Materials

Supplementary materials can be found at https://www.mdpi.com/1422-0067/20/6/1393/s1.

## Abbreviations

DEX	Dexamethasone
LPS	Lipopolysaccharide
PCA	Principal component analysis
GCs	Glucocorticoids
HPA	Hypothalamus-pituitary-adrenal
ACTH	Adrenocorticotropic hormone
BUN	Blood urea nitrogen
ALT	Alanine aminotransferase
