# Supplementary material for: Kinetics of Physiological and Behavioural Responses in Endotoxemic Pigs with or without Dexamethasone Treatment
Source: Int J Mol Sci. 2019 Mar 20;20(6):1393. doi: 10.3390/ijms20061393 (PMC6471452; doi:10.3390/ijms20061393)
Supplement: Supplementary file 1 [file ijms-20-01393-s001.pdf]

Table S1. Overview of the effect of the time point and the treatment on studied parameters.

Parameter	Unit	*p*-value			LSM ± SE							
					Saline				Dexamethasone			
		Time	Treatment	T × T	T0-3 h	T0	T0+1 h	T0+3 h	T0-3 h	T0	T0+1 h	T0+3 h
RT	°C	<.0001	0.0622	0.0579	39.21 ± 0.12^{a}	39.28 ± 0.07^{a}	39.85 ± 0.15^{b}	39.29 ± 0.28^{ab}	39.19 ± 0.12^{A}	39.26 ± 0.07^{A}	39.97 ± 0.15^{B}	40.38 ± 0.27^{B}
Cortisol	ng/ml	<.0001	0.0019	<.0001	28.46 ± 3.09^{a}	23.68 ± 2.49^{a}	79.73 ± 6.99^{a}	129.11 ± 14.35^{b}	28.68 ± 3.00^{A}	5.70 ± 2.42^{B}	41.55 ± 6.79^{A}	85.70 ± 13.94^{C}
ACTH	pg/ml	<.0001	0.0013	<.0001	25.44 ± 6.50^{a}	21.23 ± 3.19^{a}	153.72 ± 12.27^{b}	196.29 ± 21.21^{c}	31.08 ± 6.31^{A}	5.97 ± 3.10^{B}	45.34 ± 11.92^{A}	152.22 ± 20.61^{C}
Glucose	mg/dl	<.0001	0.0004	0.0032	116.02 ± 3.80^{a}	106.90 ± 3.24^{a}	109.43 ± 3.62^{a}	86.96 ± 6.49^{b}	116.67 ± 3.69^{A}	123.67 ± 3.14^{A}	134.22 ± 3.51^{B}	114.22 ± 6.30^{A}
Triglyceride	mg/dl	<.0001	0.0004	0.0008	44.77 ± 4.44^{ab}	34.94 ± 2.79^{a}	49.83 ± 3.55^{b}	74.77 ± 5.76^{c}	39.46 ± 4.31^{A}	30.46 ± 2.71^{A}	28.41 ± 3.44^{A}	38.63 ± 5.60^{A}
Lactate	mmol/l	<.0001	0.1234	0.1982	7.02 ± 0.58^{b}	6.45 ± 0.54^{ab}	4.94 ± 0.35^{a}	6.93 ± 0.48^{b}	6.26 ± 0.57^{A}	6.36 ± 0.53^{A}	4.52 ± 0.34^{B}	5.34 ± 0.46^{AB}
Creatinine	μmol/l	<.0001	0.3560	0.3451	69.32 ± 3.13^{a}	70.87 ± 3.19^{a}	70.96 ± 2.66^{a}	83.84 ± 4.20^{b}	67.75 ± 3.04^{A}	64.75 ± 3.09^{A}	69.92 ± 2.57^{AB}	77.45 ± 4.08^{B}
BUN	mg/dl	<.0001	0.9858	<.0001	4.60 ± 0.52^{a}	4.89 ± 0.56^{a}	5.20 ± 0.59^{a}	5.89 ± 0.70^{b}	3.37 ± 0.50^{A}	4.92 ± 0.54^{B}	5.72 ± 0.57^{C}	6.51 ± 0.68^{D}
ALT	U/l	0.0005	0.3575	0.3645	40.29 ± 2.72^{a}	40.82 ± 2.59^{a}	39.59 ± 2.52^{a}	36.29 ± 2.09^{b}	43.03 ± 2.64^{A}	43.26 ± 2.51^{A}	42.20 ± 2.45^{A}	41.03 ± 2.02^{A}
RBC	10^6/mm^3	0.0038	0.0051	0.0012	6.11 ± 0.15^{a}	5.99 ± 0.13^{a}	6.11 ± 0.11^{a}	6.53 ± 0.15^{b}	5.99 ± 0.15^{B}	5.57 ± 0.13^{A}	5.61 ± 0.11^{A}	5.55 ± 0.15^{A}
Hemoglobin	g/dl	0.0111	0.0015	0.0011	10.48 ± 0.24^{a}	10.28 ± 0.20^{a}	10.51 ± 0.18^{a}	11.23 ± 0.25^{b}	10.27 ± 0.23^{B}	9.58 ± 0.19^{A}	9.63 ± 0.18^{A}	9.48 ± 0.24^{A}
Hematocrit	%	0.0047	0.0016	0.0013	35.53 ± 0.82^{a}	34.81 ± 0.68^{a}	35.32 ± 0.60^{a}	38.00 ± 0.83^{b}	34.82 ± 0.79^{B}	32.44 ± 0.66^{A}	32.54 ± 0.58^{A}	32.14 ± 0.80^{A}
Leukocytes	10^3/mm^3	<.0001	0.5934	0.0069	21.66 ± 1.41^{a}	23.26 ± 1.56^{a}	11.71 ± 2.10^{b}	12.21 ± 2.60^{b}	21.21 ± 1.36^{B}	27.68 ± 1.51^{C}	13.59 ± 2.04^{A}	11.26 ± 2.52^{A}
Lymphocytes	%	<.0001	<.0001	<.0001	58.41 ± 2.11^{a}	55.45 ± 2.42^{a}	69.15 ± 3.89^{b}	56.28 ± 3.67^{a}	60.47 ± 2.04^{B}	31.04 ± 2.34^{C}	45.98 ± 3.78^{A}	40.81 ± 3.56^{A}
Platelets	10^3/mm^3	<.0001	0.8278	0.0020	430.19 ± 34.24^{ab}	494.96 ± 21.58^{a}	383.07 ± 22.37^{b}	259.60 ± 26.38^{c}	427.68 ± 33.24^{AB}	416.90 ± 20.91^{A}	412.57 ± 21.68^{A}	335.90 ± 25.58^{B}
IL-1β	Log10, pg/ml	<.0001	0.8854	0.3932	2.27 ± 0.03^{a}	2.30 ± 0.03^{a}	2.41 ± 0.03^{b}	3.07 ± 0.10^{c}	2.34 ± 0.03^{A}	2.36 ± 0.03^{A}	2.44 ± 0.03^{B}	2.89 ± 0.10^{C}
IL-6	Log10, pg/ml	<.0001	0.0864	0.1743	1.54 ± 0.02^{a}	1.58 ± 0.02^{a}	2.29 ± 0.10^{b}	3.28 ± 0.18^{c}	1.56 ± 0.02^{A}	1.59 ± 0.02^{A}	1.99 ± 0.10^{B}	2.88 ± 0.18^{C}
IL-10	Log10, pg/ml	<.0001	0.2818	0.4156	1.98 ± 0.09^{a}	2.04 ± 0.08^{a}	2.54 ± 0.07^{b}	2.36 ± 0.08^{c}	2.19 ± 0.08^{AB}	2.15 ± 0.08^{A}	2.54 ± 0.07^{C}	2.42 ± 0.08^{B}
TNF-α	Log10, pg/ml	<.0001	0.1419	0.0530	1.35 ± 0.04^{a}	1.41 ± 0.05^{a}	3.69 ± 0.27^{b}	2.78 ± 0.17^{c}	1.39 ± 0.04^{A}	1.30 ± 0.04^{A}	3.20 ± 0.26^{B}	2.41 ± 0.16^{C}

Single underline highlights significant main effects at $p < 0.05$. Double underline highlights significance between saline and dexamethasone groups at the same time point ($p < 0.05$). Within each group, superscript highlights the significance among different time points; same superscript highlights non-significance ($p > 0.05$); different superscript highlights significance ($p < 0.05$); lowercase letter highlights the significance for saline group, capital letter highlights the significance for DEX group. T × T: time point-by-treatment interaction. LSM: least-squares means. RT: rectal temperature. ACTH: adrenocorticotropic hormone. BUN: blood urea nitrogen. ALT: alanine aminotransferase. RBC: red blood cells.

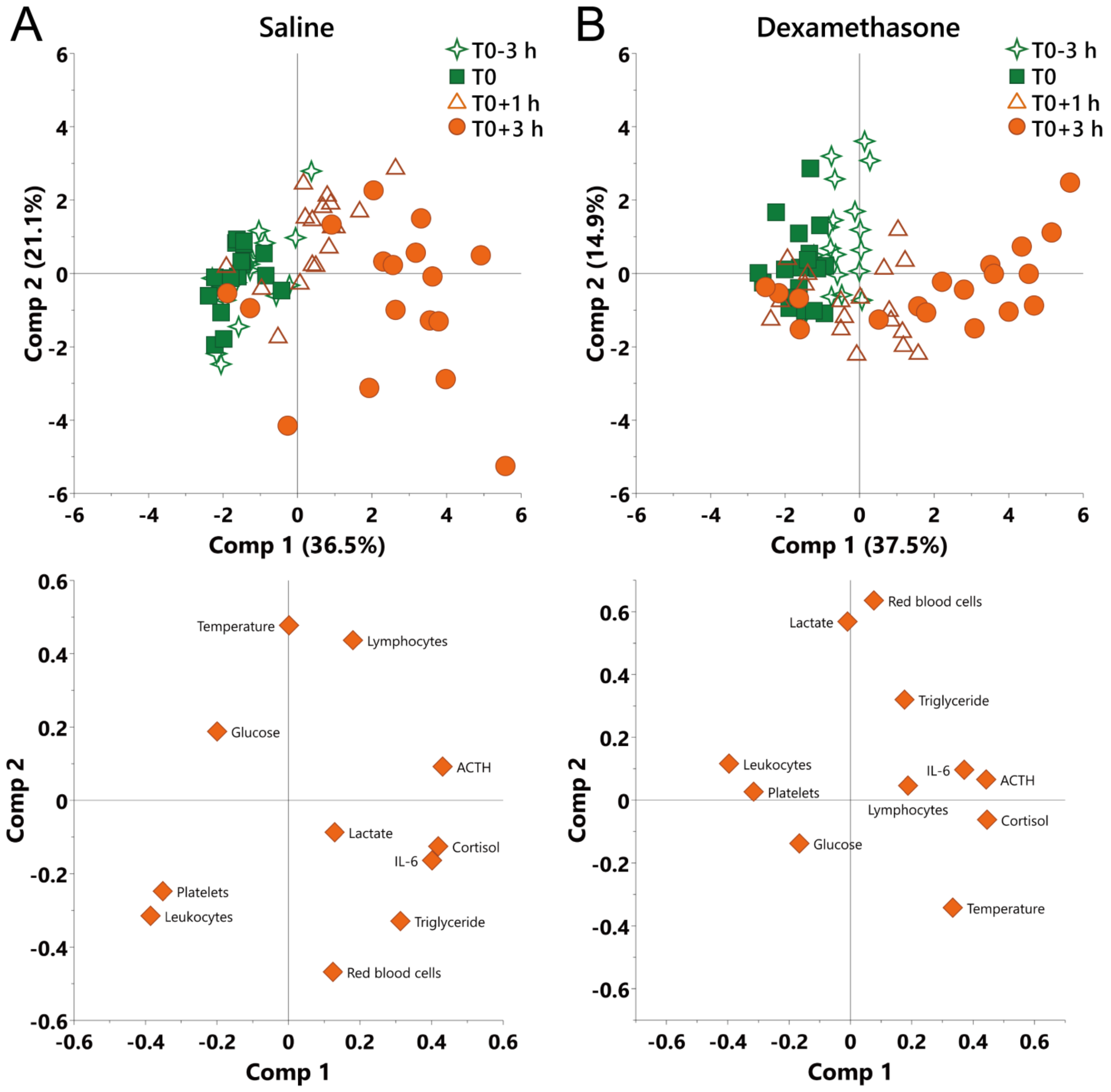

Figure S1. Principal component analysis (PCA) for time-depending overall effect of LPS on pigs with or without pretreatment of DEX. (**A**) Pigs pretreated with saline; and (**B**) pigs pretreated with DEX. Loading of the parameters on component 1 and 2 are visualized below the PCA score plot to show the contribution of parameters to the group separation. Comp, component.
